# A characterization of postzygotic mutations identified in monozygotic twins

**DOI:** 10.1002/humu.23586

**Published:** 2018-07-18

**Authors:** Klaasjan G. Ouwens, Rick Jansen, Bas Tolhuis, P. Eline Slagboom, Brenda W.J.H. Penninx, Dorret I. Boomsma

**Affiliations:** ^1^ Department of Biological Psychology VU University Amsterdam Amsterdam The Netherlands; ^2^ Genalice Core BV Nijkerk The Netherlands; ^3^ Department of Psychiatry VU University Medical Center Amsterdam The Netherlands; ^4^ Department of Molecular Epidemiology Leids Universitair Medisch Centrum Leiden The Netherlands

**Keywords:** genetics, mutations, next‐generation sequencing, postzygotic mutations, somatic mosaicism

## Abstract

Postzygotic mutations are DNA changes acquired from the zygote stage onwards throughout the lifespan. These changes lead to differences in DNA sequence among cells of an individual, potentially contributing to the etiology of complex disorders. Here we compared whole genome DNA sequence data of two monozygotic twin pairs, 40 and 100 years old, to detect somatic mosaicism. DNA samples were sequenced twice on two Illumina platforms (13X and 40X read depth) for increased specificity. Using differences in allelic ratios resulted in sets of 1,720 and 1,739 putative postzygotic mutations in the 40‐year‐old twin pair and 100‐year‐old twin pair, respectively, for subsequent enrichment analysis. This set of putative mutations was strongly (*p* < 4.37e–91) enriched in both twin pairs for regulatory elements. The corresponding genes were significantly enriched for genes that are alternatively spliced, and for genes involved in GTPase activity. This research shows that somatic mosaicism can be detected in monozygotic twin pairs by using allelic ratios calculated from DNA sequence data and that the mutations which are found by this approach are not randomly distributed throughout the genome.

## 1 INTRODUCTION

When the DNA of a person does not encompass the same sequence in every cell of the body, but contains de novo postzygotic genetic mutations in a fraction of the cells only, the person is considered a mosaic. This is different from genetic chimerism, where a single organism is composed from different zygotes, for example, after transplacental exchange between mother and child or between twins (vanDijk, Boomsma, & deMan, 1996). In the literature, mosaicism has been described in phenotypically discordant monozygotic twin pairs (Zwijnenburg, Meijers‐Heijboer, & Boomsma, 2010) and postzygotic mutations are considered as a possible cause for such twin discordance. Recent research found that a fraction of presumed germline de novo mutations are actually either postzygotic or inherited as a consequence of low‐level mosaicism in one of the parents (Acuna‐Hidalgo et al., 2015; Rahbari et al., 2016), and that early postzygotic mutations could account for a substantial proportion of de novo single nucleotide variants (SNVs) in the genome of an individual (Dal et al., 2014). Postzygotic mosaic mutations are usually associated with cancer development (Abyzov et al., 2017; Biesecker & Spinner, 2013; Buntinx, Campbell, & van den Akker, 2014; Cohen, Wilson, Trinh, & Ye, 2015; Forsberg, Absher, & Dumanski, 2013; Iourov, Vorsanova, & Yurov, 2010; Jacobs et al., 2012; Laurie et al., 2012), but they are also an important confounder in medical genetic testing (Forsberg, Gisselsson, & Dumanski, 2017). A recent study suggests that somatic mosaicism in the brain might represent a potential mechanism contributing to neuronal diversity and the etiology of neuropsychiatric disorders (McConnell et al., 2017). The number of clonal SNVs has been estimated at 1,000–1,500 per neuronal genome which are enriched in coding exons (Lodato et al., 2015).

Monozygotic (MZ) twins are often called identical twins, since they are presumed to have no differences at the level of the DNA sequence. MZ twins arise from one fertilized oocyte, and there is a chance of a somatic mutation at each subsequent mitosis. The moment in life at which these mutations occur determines whether they are present in both twins, in only one twin, or even in only a fraction of the cells in one twin (Martin, Boomsma, & Machin, 1997). Throughout life, limitations in somatic cell maintenance lead to accumulation of mutations. Aging is thought to be a consequence of this accumulation (Jacobs et al., 2012; Veitia, Govindaraju, Bottani, & Birchler, 2017). Since mechanisms of cellular and molecular aging are inherently stochastic, this will cause MZ twins to diverge (Kirkwood, 2005).

An example of large‐scale genomic mosaicism is the phenomenon of early postzygotic mitotic nondisjunction resulting in MZ twins having different numbers of chromosomes. There have been documented cases where twins are mosaic 45,X/46,XY and discordant for phenotypic sex (Reindollar, Byrd, Hahn, Haseltine, & Mcdonough, 1987). The same is possible for MZ discordances for autosomal trisomies (e.g., Down syndrome [MIM: 190685]). Compared to germline aneuploidies, many more mosaic aneuploidies have been found to be compatible with life, including monosomy 7 and 18, and trisomies 7, 8, 9, 12, 14, 15, 16, 17, and 20 (Machin, 1996). This is an indication that postzygotic mutations causative of heritable disease may result in a milder phenotype. Small‐scale genomic mosaicism is also possible, including single nucleotide substitutions. For example, somatic mosaicism for a mutation in the *COL4A5* gene (HGNC: 2207) is the cause of a milder phenotype of male Alport syndrome (Krol et al., 2008). Similarly, a somatic mutation partially rescuing a child with Hutchinson–Gilford progeria syndrome was recently reported (Bar et al., 2017).

Given the number of mitoses required for human development, it is plausible that every human has some cells harboring a mutation causative of genetic disease (Behjati et al., 2014; Gong, Gu, & Woodruff, 2005; Iourov et al., 2010; Seshadri, Kutlaca, Trainor, Matthews, & Morley, 1987). However, the level of mosaicism can be very low (i.e., with a postzygotic mutation visible in only a small subset of somatic cells) which will make mosaicism difficult to detect, especially using Sanger sequencing (Beicht et al., 2013). Next‐Generation Sequencing (NGS) has facilitated faster sequencing with a lower per‐base cost; however, restricted budgets still limit the maximum read depth of available data. While a medium read‐depth (i.e., below 30X) could be sufficient for sensitivity and specificity regarding constitutional mutations, this might not be the case for detecting mosaicism.

Ye et al. reported on a sequencing project in DNA samples from peripheral blood of two MZ twin pairs of differing ages (40 and 100 years old) using two different sequencing and variant calling pipelines (Ye et al., 2013). One pipeline used Illumina 40X sequencing, alignment with Burrows–Wheeler Aligner (BWA) (H. Li & Durbin, 2009) and compared the four nucleotide base counts per genomic location between co‐twins using the CaVEMAN pipeline developed by the Wellcome Trust Sanger Institute (Stephens et al., 2012). The second pipeline used Complete Genomics whole‐genome sequencing, where between‐twin variant calls were determined using the Complete Genomics tumor variant calling tool (Drmanac et al., 2010). Intersecting the large numbers of putative mosaic mutations for each platform resulted in 13 and 17 potential postzygotic mutations occurring in the 40‐year‐old and 100‐year‐old twin pairs, respectively. After validation with Sanger sequencing, Ye et al. found no somatic mutations in the 40‐year‐old twin pair, and eight validated somatic mutations in the older twin pair, consistent with the theory of aging as accumulation of somatic variants (Ye et al., 2013). However, this might be a conservative estimate of the true rate of mosaicism since mutations might have been missed by the sequence alignment software or by applying tumor‐specific analysis software, or may have been detected by one approach and not the other, thereby leading to a very small intersection of variants that were detected by both platforms.

**Figure 1:**
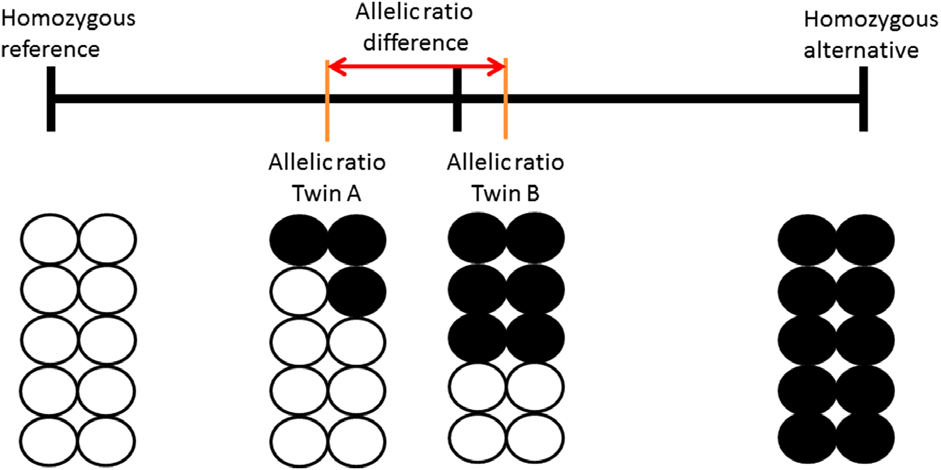
The result of a possible postzygotic mutation during early development in a monozygotic twin pair. Circles represent somatic cells, with cells containing a postzygotic mutation in black. A locus that was heterozygous before the twinning event may show different allele fractions between co‐twins posttwinning. NGS variant calling software would generally call both co‐twins heterozygous at this locus

Here we use an alternative method for detecting postzygotic mutations in the same two twin pairs studied by Ye et al., analyzing measurements from two Illumina sources, at read depths 13X and 40X as opposed to Ye et al. (2013). We calculated for each locus in each person the allelic ratio: the fraction of alternative read counts as part of the total read depth at that locus. This is similar to deviation from genotype‐expected b‐allele frequency (*B*_dev_), which is considered for detecting larger structural mosaic events such as copy number variations (King et al., 2017). With this method, having only reference reads generates a zero value, having 50% alternative reads will get value 0.5 (being heterozygous) and having only alternative reads will get value 1 (see Figure 1). However, any value between these values is also possible (e.g., having 30% alternative reads at a locus). This is different from standard variant calling, where loci are called either heterozygote or homozygote without any in‐between values. For each locus, we compared this quantitative measure between the co‐twins from the same pair and for the two sequences from the same individual. The larger the difference between the allelic ratios, the more the co‐twins differ from each other at that locus, or the twin from herself between the 13X and 40X reads. Any difference between co‐twins will represent a posttwinning event. In contrast, any differences within an individual are likely to represent noise. Mutations arising from parental germline mosaicism or during pretwinning stages will be present in both co‐twins and therefore not detected by our method. Using this technique, we were able to identify multiple putative mosaic sites, which were then characterized in terms of position and function.

## 2 MATERIALS AND METHODS

### 2.1 Next‐generation sequencing data measurements and analysis

One MZ twin pair aged 40 years was selected from the Netherlands Twin Register (Willemsen et al., 2013), and one MZ twin pair aged 100 years was selected from the Leiden Longevity Study (Westendorp et al., 2009). Both pairs were female. Data collection was approved by the Medical Ethics Committee of the Leiden University Medical Center and the Medical Ethics Committee of the VU Medical Centre, Amsterdam. Informed, written consent was obtained from the twins participating in the study. DNA was extracted from whole blood and sequenced using the Illumina HiSeq 2000 platform (91‐basepair [bp] paired‐end reads) at read depth 13X with the library preparation protocol developed for the Genome of the Netherlands project (Boomsma et al., 2014). For the same samples, 100‐bp paired‐end reads were generated using Illumina GAIIx instruments at read depth 40X using the manufacturer standard protocol and library as used in Ye et al. (2013). Including the Illumina 13 × 91 bp paired‐end reads should increase the reliability of the read alignments, increasing the probability of placing a read with nonreference variants at the correct genomic location. Using a combination of two different sequencing platforms should reduce the influence of errors from PCR and sequencing.

Both Illumina‐based NGS datasets were aligned to GRCh37 using gaMap 2.4.0 by Genalice. Variant calling was done with gaVariant 2.4.0. Both tools by Genalice were recently benchmarked against PEMapper/PECaller, BWA/GATK and Isaac (Pluss et al., 2017) showing comparable sensitivity (> 0.998) to BWA/GATK and outperforming PEMapper/PECaller and Isaac. The calling process included removal of PCR duplicates and removal of low‐quality bases and reads, and local realignment to reduce false positive variant calls. Variant calling was optimized by applying a softclip to reads, and by filtering on quality‐ and coverage‐induced noise levels (see https://www.genalice.com/download-whitepapers/). We restricted the analysis to high‐confidence regions of the genome (Rosenbloom et al., 2015; Zook et al., 2014), a collection of regions identified by the Genome In A Bottle consortium (GIAB). By integrating and arbitrating between 14 data sets from five sequencing technologies, seven read mappers and three variant callers, GIAB published regions in the genome where systematic sequencing errors, local alignment errors, and mapping errors have minimal influence while minimizing bias toward any individual sequencing platform. In practice, about 23% of the genome is discarded for displaying systematic sequencing errors or mapping difficulties (e.g., due to many simple repeats). In addition, we omitted repetitive regions of the genome using the University of California Santa Cruz (UCSC) RepeatMasker track (Karolchik et al., 2004). To ensure validity of our variant calling methods, we compared the resulting Variant Call Format file (VCF) with the results from a Burrows‐Wheeler aligner/Genome Analysis Toolkit (BWA/GATK) pipeline following manufacturers’ best practices (Van der Auwera et al., 2013). Using results from BWA/GATK as a baseline, we found a mean sensitivity, specificity, and F‐ratio of 0.9858, 0.9811, and 0.9834 respectively (see Supporting Information Table S1). Further downstream analysis was done using a combination of custom shell, Perl, and R scripts. Loci where both co‐twins were homozygous for the reference allele were discarded. These sites have, per definition, not one single reliably detected difference between twins and as a result do not contain information on possible postzygotic mutations. For each co‐twins, the allelic ratio was calculated: the fraction of the alternative read count compared to the total read count. Subsequently, the difference in allelic ratio was calculated for each locus between co‐twins. This resulted in a number between –1 and 1, where 0 indicates no difference between co‐twins and higher absolute numbers indicate larger differences between co‐twins (as shown in Figure 1).

### 2.2 Statistical analysis

**Figure 2:**
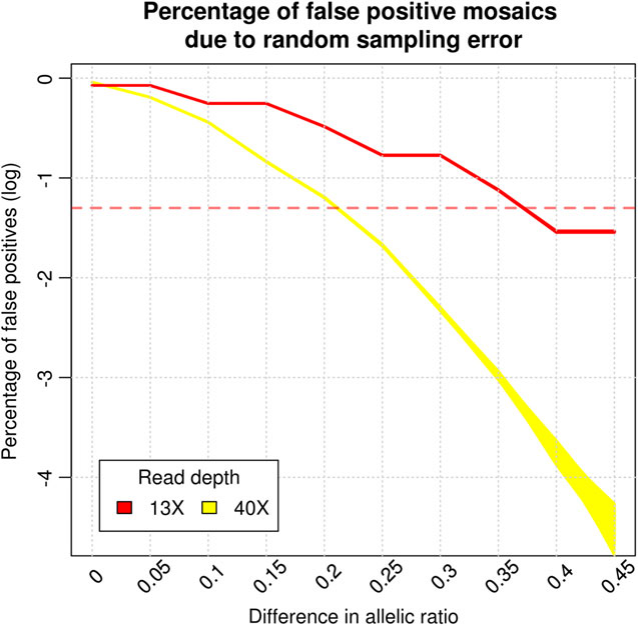
The chance of finding a postzygotic mutation because of sampling error in 40X and 13X data, based on 1,000 simulations of 893,581 heterozygote sites. The red dotted line indicates 5% false positives

Under the null hypothesis of absence of mosaic loci, it is still possible to find false positive putative mosaic sites at heterozygous loci due to random sampling. This is in addition to other sources of false positives including sequencing, PCR and mapping errors. Even though both co‐twins may be heterozygous for a locus, there is still a chance that the ratio of alternative alleles deviates from 50%. To ascertain the false positive rate because of this sampling noise, we simulated two binomial distributions, with 40 observations (the read depth) and chance of success 0.5 and calculated the number of times a difference between co‐twins was observed. We performed 1,000 simulations of 893,581 loci, the average number of heterozygous loci we analyzed per twin pair. We computed that due to random sampling we could expect 0.36% of these loci to have a difference in allelic ratio higher than 0.25 in both 40X and 13X data (see Figure 2 and Supporting Information Table S2).

We performed permutations to test for overabundance of postzygotic mutations within short distance to each other, within twin pairs and between twin pairs. For each permutation (*N* = 1,000), we extracted random loci from the reference set (*n* = 1,720 and *n* = 1,739 for the 40‐year‐old twin pair and the 100‐year‐old twin pair, respectively) and computed the number of loci with a distance smaller than the threshold, which we used as null distribution for the permutation test. Finally, loci were annotated using Variant Effect Predictor (VEP) (McLaren et al., 2016) and an enrichment test was performed using Fisher‐exact tests. A gene‐based enrichment analysis was performed with DAVID v6.8 (Huang da, Sherman, & Lempicki, 2009). For an additional validation of the enrichment analysis, we compared 13X and 40X sequencing data sets from each individual and tested whether loci with an allelic ratio difference were enriched for intronic regions. If differences within an individual truly represent noise in the data, we predict that we will not observe any enrichments.

## 3 RESULTS

### 3.1 Next‐generation sequencing data

**Figure 3:**
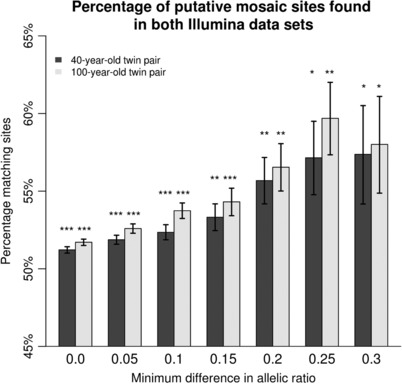
The fraction of putative mosaic sites found in both Illumina sets for both twin pairs. Asterisks indicate significance of binomial test of matching sites versus nonmatching sites. *p < 1e–5; **p < 1e–10, ***p < 1e–20

DNA sequencing was measured in an MZ twin pair aged 40 years and an MZ twin pair aged 100 years. DNA was extracted from whole blood and sequenced at read depth 13X and at read depth 40X. Variant calling was restricted to high‐confidence regions of the genome, and repetitive regions were omitted (see Materials and Methods). After discarding loci where both co‐twins were homozygous for the reference allele, and taking the intersection of loci found from both sequencing sets, 881,298 and 905,864 single‐nucleotide loci were left for analysis for the 40‐year‐old and 100‐year‐old twin pair, respectively (see Supporting Information Table S3 for a flowchart of several filtering steps). We calculated for each locus in each person the allelic ratio: The fraction of alternative read counts as part of the total read depth at that locus and for each twin pair the allelic ratio difference at each site. This was done for both sequencing sets. Subsequently, we looked at the number of matching loci, here defined as sites where the allelic ratio difference has the same sign, according to both sequencing platforms. To increase the number of matching loci, we employed two filtering steps. We applied a threshold for minimum allelic ratio difference in both 40X and 13X data. We subsequently checked the percentage of matching loci at several thresholds and tested if the number of matching loci was significantly higher than the number of nonmatching loci using a binomial test. Second, we found that conditioning on loci where one co‐twin is clearly not mosaic (having an allelic ratio that differs less than 0.05 from 0, 0.5, or 1) also improves the percentage of matching loci. At minimum allelic ratio difference zero, this clear‐call filter reduced the number of sites in our set from 881,298 to 226,945 (40‐year‐old twin pair) and from 905,864 to 225,010 (100‐year‐old twin pair). Increasing the threshold for allelic ratio differences up to 0.25 and using the clear‐call filter increased the percentage and significance of matching loci for both twin pairs (see Figure 3). Therefore, we applied the clear‐call filter and a threshold of 0.25 for both twin pairs. This resulted in 57.15% matching loci in the 40‐year‐old twin pair *(N* = 1,720, *p *= 3.274e–9) and 59.69% matching loci in the 100‐year‐old twin pair (*N* = 1,739, *p* = 6.355e–16). Out of these two sets, 19 loci were found to be simultaneously putatively mosaic in both twin pairs (see Supporting Information Table S3 for a detailed overview of all filtering steps).

Our choice for 0.25 as a threshold for minimal difference in the allelic ratio was supported by our simulations. In our data, the percentage of loci with an allelic ratio higher difference than 0.25 was 0.90% (40‐year‐old twins) and 0.89% (100‐year‐old twins; see Supporting Information Table S3). This difference was statistically significant (*p* < 0.001, 1,000 permutations) from what we would expect due to random sampling (0.36%). Sampling heterozygote loci influencing erroneous reporting of mosaics shows that setting a threshold for allelic ratio difference of > 0.25 led to a maximum of 2% false positive mosaics due to random sampling (see Figure 2). Loci identified in both the 13X and 40X data with the same direction of effect of allelic ratio difference are more likely to be true positives. Likewise, nonmatching loci, having opposite‐signed effects, are more likely to be false positives. Thus, the difference between the percentage of matching loci (57.15% and 59.69%) and the percentage of nonmatching loci can be considered as an estimate of the percentage of true positives (14.3% and 19.38%, respectively). Note that this estimate only holds for differences identifiable by both 13X and 40X platforms.

### 3.2 Enrichment analyses

Within our respective sets of 1,720 and 1,739 putative mosaic mutations, we found enrichment for mosaics that are within 101–500, 501–1,000, 1,001–5,000, and 5,001–10,000 bp from each other (all nominally significant; see Supporting Information Table S4). Between the twin pairs, we also found that postzygotic mutations seem to cluster in hotspots with genetic distances up to 10,000 bp (10 pairs at 501–1,000 bp, 7 pairs at 501–1,000 bp, 40 pairs at 1,001–5,000 bp, 33 pairs at 5,001–10,000 bp, all *p* < 0.001; see Supporting Information Table S5). This enrichment gets less strong for larger genetic distances. We used VEP to annotate the results of putative mosaic mutations, and tested with a Fisher's exact test whether the 14 single‐nucleotide polymorphism (SNP) categories provided by VEP were significantly enriched compared to our full list of heterozygous variants. From the 14 categories, nine were enriched in the 40‐year‐old twin pair and 10 were enriched in the 100‐year‐old twin pair. Remarkably, for both twin pairs the strongest enrichments were in the categories *regulatory elements* and *5′ untranslated regions* (*p* < 4.37e–91, *p* < 7.94e–33, Table 1). Post hoc analysis showed that the significance of this enrichment increases with the applied filter steps (Supporting Information Table S3). Additionally, we tested for enrichment of putative mosaic mutations in two genes, *DNMTA3* (HGNC:2978) and *TET2* (HGNC:25941), both linked to clonal expansion of hematopoietic stem cells. Somatic mosaicism in these genes was reported to be common in the elderly (Acuna‐Hidalgo et al., 2017; van den Akker et al., 2016; Zink et al., 2017). We found a slight enrichment for low‐mutational ratio mosaicism in these genes in both twin pairs (minimal difference in allelic ratio threshold > 0.05, 40‐year‐old twin pair *p* = 0.0023, 100‐year‐old twin pair *p* = 0.0031; Supporting Information Table S3). For an additional validation of the enrichment analysis, we compared two sequencing runs of the same individual using the same procedure as was used for the co‐twins. We identified loci with allelic ratio differences in these sequence runs and tested whether these loci were enriched for intronic regions. The only significant enrichment was found when comparing the 40X data between co‐twins, but not when comparing different sequencing runs of the same person. This indicates that the identified putative postzygotic mutations may contain a high number of false positives, but these false positives are unlikely to drive the reported enrichment (see Supporting Information Tables S6 and S7).

**Table 1:** Enrichment of variant types in the set of putative postzygotic mutations

	40‐year‐old twin pair ARD > 0	40‐year‐old twin pair ARD > 0.25	Enrichment *p*‐value	Enrichment *p*‐value, FDR‐corrected	100‐year‐old twin pair ARD > 0	100‐year‐old twin pair ARD > 0.25	Enrichment *p*‐value	Enrichment *p*‐value, FDR‐corrected
Total variants	226,945	1,720			225,010	1,739		
Intronic	55.4%	59.7%	*9.46e–05*	1.66e–04	55.2%	59.6%	*5.39e–05*	*1.26e–04*
Intergenic	35.1%	24.5%	1	1	35.4%	24.6%	1	1
Modifier	99.7%	99.8%	0.338	0.364	99.8%	99.7%	0.840	0.905
Low impact	0.9%	2.6%	*1.79e–09*	5.01e–09	0.9%	2.0%	*1.80e–05*	*5.04e–05*
Moderate impact	0.6%	1.5%	*1.57e–05*	3.14e–05	0.6%	1.3%	*5.15e–04*	*7.99e–04*
High impact	0.03%	0.1%	0.0978	0.114	0.03%	0.1%	0.0915	0.107
Noncoding	32.4%	35.6%	*1.52e–03*	2.13e*–*03	32.1%	35.8%	*2.80e–04*	*5.60e–04*
Synonymous	0.7%	1.5%	*2.06e–04*	3.20e*–*04	0.7%	1.4%	*5.71e–04*	*7.99e–04*
Missense	0.6%	1.5%	*1.57e–05*	3.14e*–*05	0.6%	1.3%	*5.15e–04*	*7.99e–04*
Regulatory	9.1%	25.7%	*4.37e–91*	6.12e*–*91	9.1%	25.8%	*4.66e–93*	*6.52e–92*
TF binding	0.7%	2.9%	*8.10e–18*	3.78e*–*17	0.7%	2.7%	*7.68e–15*	*2.69e–14*
Protein coding	49.2%	57.6%	*4.52e–13*	1.582e*–*12	49.4%	59.1%	*1.42e–16*	*6.63e–16*
3′ UTR	1.7%	2.4%	0.0118	0.015	1.7%	2.8%	*6.75e–04*	*8.59e–04*
5′ UTR	0.4%	4.8%	*1.15e–57*	8.05e*–*57	0.4%	3.3%	*7.94e–33*	*5.56e–32*
1 KG median	31.6	32.7			31.3%	33.9		
1 KG mean	34.8	35.6			34.6%	36.1		
Freq < 0.1	16.0%	13.4%			16.6%	14.4%		
Percentage in 1000G	96.6%	82.6%			96.7%	82.5%		

*Notes*. ARD = allelic ratio difference; FDR = false discovery rate. For an explanation of the different annotation terms, see https://www.ensembl.org/info/genome/variation/predicted_data.html#consequences. *p*‐values in italics are significant after correction for multiple testing.

Using DAVID, we tested whether genes where a mosaic mutation was found (*N* = 1023 for the 40‐year‐old twins, *N* = 1,055 for the 100‐year‐old‐twins) were enriched in functionally related gene groups. After false discovery rate (FDR) correction, nine and 16 gene groups were found to be significantly enriched in the 40‐year‐old and 100‐year‐old twin pairs respectively (see Table 2 and Supporting Information Tables S8 and S9). Of these gene groups, five overlapped: alternative splicing, splice variant, polymorphism, cell junction, sequence variant, and GTPase activity. The latter was annotated for the 40‐year‐old twin pair as GO‐term *Positive regulation of GTPase activity*, while it was annotated for the 100‐year‐old twin pair as UniProt‐term *GTPase activator activity*. GTPase activating proteins are essential modulators of the biological activity of guanine nucleotide binding proteins (G‐proteins). G‐protein‐coupled receptors are crucial players in tumor growth and metastasis (Dorsam & Gutkind, 2007).

**Table 2:** Significant results from functional annotation using DAVID

		40‐year‐old twin pair		100‐year‐old twin pair	
Database	Term	*p*‐value	FDR *p‐*value	*p*‐value	FDR *p*‐value
UP	Alternative splicing	7.47e*–*18	1.05e*–*14	2.43e*–*22	3.45e*–*19
UP	Splice variant	1.09e*–*11	1.92e*–*08	7.73e*–*19	1.39e*–*15
UP	Polymorphism	3.55e*–*07	4.992647e*–*04	1.19e*–*07	1.69e*–*04
KEGG	Inflammatory mediator regulation of TRP channels	1.11e*–*06	1.45e*–*04	n.s.	n.s.
GO	Intracellular signal transduction	1.53e*–*06	2.75e*–*03	n.s.	n.s.
GO	Positive regulation of synapse assembly	7.95e*–*06	0.0143	n.s.	n.s.
GO	Positive regulation of GTPase activity	1.01e*–*05	0.0182	n.s.	n.s.
UP	Cell junction	1.50e*–*05	0.0211	8.96e*–*07	1.27e*–*03
UP	Sequence variant	1.36e*–*05	0.0241	1.19e*–*05	0.0214
UP	Synapse	n.s.	n.s.	1.74e*–*06	2.47e*–*03
UP	Ion channel	n.s.	n.s.	2.00e*–*06	2.84e*–*03
UP	Epidermal growth factor‐like domain	n.s.	n.s.	5.35e*–*06	8.92e*–*03
INTERPRO	Axon guidance	n.s.	n.s.	5.99e*–*06	0.011
GO	EGF‐like domain	n.s.	n.s.	1.26e*–*05	0.0178
UP	Ig‐like C2‐type 5	n.s.	n.s.	9.95e*–*06	0.0179
UP	GTPase activator activity	n.s.	n.s.	1.40e*–*05	0.0220
GO	Membrane	n.s.	n.s.	1.73e*–*05	0.0245
UP	Metal‐binding	n.s.	n.s.	1.79e*–*05	0.0254
UP	Metal ion binding	n.s.	n.s.	2.45e*–*05	0.0386
GO	EGF‐like 2	n.s.	n.s.	2.26e*–*05	0.0405

*Notes*. FDR = false discovery rate; GO = Gene Ontology; KEGG = Kyoto Encyclopedia of Genes and Genomes; UP = UniProt.

## 4 DISCUSSION

By using the difference in alternative allele ratio in MZ twin pairs as a measure for mosaicism, we identified and annotated a set of 1,720 and 1,739 loci containing putative mosaic mutations in a 40‐year‐old MZ twin pair and a 100‐year‐old MZ twin pair, respectively. The number of putative mutations identified in blood‐derived DNA, although with a high false positive rate, is high compared to an earlier approximation of postzygotic mutations, which found that each individual carries about 300 postzygotic mutations also using blood‐derived DNA (R. Li et al., 2014). However, since sequencing was done with two separate libraries (40X and 13X) and we limited ourselves to high‐confidence regions of the genome, we were able to identify preferential enrichment of postzygotic mutations. The differences between twins we identified point to clustering of postzygotic mutations around hotspots up to 10 Mb in size. We found similar patterns of strong enrichments for variant types and gene sets in both twin pairs, suggesting that postzygotic mutations follow a nonrandom pattern, confirming recent findings (Vattathil & Scheet, 2016). The enrichment of regulatory elements suggests a relevant role for mosaicisms. We replicated the finding by Lodato (2015) that coding exons are enriched for postzygotic mutations (Lodato et al., 2015). The enrichment we found for mosaicism in genes associated with GTPase activity, involved in tumor growth, is in line with recent findings that larger‐scale genomic mosaicisms in genes are associated with cancer (Laurie et al., 2012; Machiela et al., 2015; Vattathil & Scheet, 2016). The continuing discovery of even more cases of mosaicism provides much‐needed insights into postzygotic mutational signatures (see, e.g., Ju et al. 2017; Martincorena and Campbell, 2016). The current research cannot speak to the role of postzygotic mutations in disease pathogenesis, as it was based on results from healthy MZ twins. The literature suggests that notion of one person not having the same genome in every cell throughout the body is of important clinical relevance as mosaicism is involved in diseases that would be lethal in constitutional state, as well as in organ‐specific diseases since mosaicism can remain site‐specific (Biesecker & Spinner, 2013).{Biesecker, 2013 #45} In a 115‐year‐old woman, somatic mutations detected in blood were not detected in other tissues or tumor tissue, indicating that these mutations were not derived from tumor cells (Holstege et al., 2014). Mosaicism has been observed in several Mendelian diseases, for example, as previously mentioned in Alport Syndrome, where somatic mosaicism resulted in an unusually mild phenotype (Bruttini et al., 2000; Krol et al., 2008). However, the level of mosaicism does not necessarily correlate with the severity of clinical manifestation, and mosaicism may even not have any visible effects (Cohen et al., 2015). Future research should take advantage of new technologies, for example, single cell sequencing, for high‐resolution detection and localization of genetic mosaicism.

In spite of the high number of false positives in the identified mutations, these false positives are unlikely to be preferentially enriched in functional elements or gene pathways, as was confirmed by additional analysis comparing 13X and 40X measurements from the same samples. We are aware that by selecting mutations that are present both in 40X and 13X data we may discard some true positive data present in 40X but not in 13X data. However, this removes more false positives than true positives. This is exemplified with the enrichment analyses, which show stronger effects after more stringent filtering. In three of the eight loci previously validated through Sanger sequencing (Ye et al., 2013), we found evidence in our data for mosaicism. Out of the 22 false‐positive mutations in Ye et al. that were shown to be false positives by Sanger sequencing, we identified 21 true‐negative and one false‐positive indication of mosaicism (see Supporting Information Table S10).

Our stringent filtering might also explain why we do not see age differences in the number of mosaic mutations. Age‐related somatic mosaicism results in very low mutational ratios (Laurie et al., 2012), whereas higher mutational ratios should occur only early after fertilization or by clonal expansion. This suggests that the mutations we extracted in our procedure likely occurred early in life, instead of being accumulated with age. It is important to note that postzygotic mutations resulting in differences in allelic ratio between MZ twins cannot be a result of parental germline mosaicism, but rather occur during embryogenesis. Parental mosaicism would result in mutations in both co‐twins, which would not result in a difference in allelic ratio between twins and would therefore not be detectable by our method.

Although Huang et al. mention that accurate identification of postzygotic mutations provides insights into finding the “missing heritability” (Huang et al., 2014), the number of expected postzygotic mutations is so low that the impact on heritability estimates in twin studies is negligible. Twin‐based heritability is based on the assumption that the additive genetic correlations of MZ and dizygotic (DZ) twins equal *r*_MZ_ = 1 and *r*_DZ_ = 0.5, respectively. If a model with *r*_MZ_ = 1 and *r*_DZ_ = 0.5 is fitted to twin data, while both *r*_MZ_ and *r*_DZ_ are marginally lower due to mosaicism, the estimate of the additive genetic variance will be only marginally biased. For instance: let mosaicism cause the *r*_MZ_ to be 0.99 and *r*_DZ_ to be 0.495, and let the true additive genetic variance be 0.35. The misspecified model (assuming *r*_MZ_ = 1 and *r*_DZ_ = 0.5) would then yield an estimate of 0.3465. The true rate of mosaicism, and therefore its contribution to the “missing heritability,” is many magnitudes lower. While the effects on the heritability are expected to be negligible, this does not rule out that nonheritable genetic variation may be an important factor in the development of sporadic diseases (Forsberg et al., 2013).

In this paper, we showed the value of the monozygotic twin design as a method to identify mosaicism. Even with a limited sample size, we established with this design that mosaic mutations are not randomly distributed across the genome but rather are highly enriched for specific genomic hotspot locations, transcript location, and gene groups.

Klaasjan Ouwens is financially supported by the EMGO Institute for Health and Care Research (EMGO+). We acknowledge BBRMI‐NL (NWO 184.021.007).

## CONFLICTS OF INTERESTS

Bas Tolhuis is a paid employee of Genalice Core BV. Klaasjan Ouwens is an embedded Ph.D. candidate at Genalice Core BV.

## Supporting information

Supporting Information Table S1: Results of comparing alignment and variant calls from Genalice tools with BWA‐GATK according to best practices.
Supporting Information Table S2: Permutation test of putative mosaicism resulting from random sampling heterozygote loci. Heterozygote sites were simulated by sampling from a binomial distribution, with the number of trials defined as the read depth (40X and 13X, respectively). 1000 permutations resulted in a number of loci that would erroneously be identified as a putative post‐zygotic mutation.
Supporting Information Table S4: Enrichment testing for within‐pair local clustering of putative post‐zygotic mutations. From the original set of 226945 (40‐year‐old twin pair) and 225010 (100‐year‐old twin pair) heterozygote loci before filtering, we performed 1000 permutations of sampling 1720 (40‐year‐old twin pair) and 1739 (100‐year‐old twin pair) loci. Subsequently we counted the number of times we saw more occurrences of post‐zygotic mutations within a window of distance and compared these to the selected set of putative post‐zygotic mutations.
Supporting Information Table S5: Enrichment testing for between‐pair local clustering of putative post‐zygotic mutations. From the original set of 226945 (40‐year‐old twin pair) and 225010 (100‐year‐old twin pair) heterozygote loci before filtering, we performed 1000 permutations of sampling 1720 (40‐year‐old twin pair) and 1739 (100‐year‐old twin pair) loci and counted the number of post‐zygotic mutations found within a specific genetic distance (ranging from 101 basepairs (bp) to 10,000 bp in both twin pairs.
Note that the number of post‐zygotic mutations where a post‐zygotic mutation was found nearby in the other twin pair was higher than was expected by chance (indicating genetic hotspots for mosaicism). This enrichment effect is stronger for smaller distances (indicated by the fold change in the last column).
Supporting Information Table S6: Number of putative mosaic loci (allelic ratio difference above 0.25) when comparing co‐twins of the same sequencing platform (left) and when comparing data from different sequencing platforms of the same co‐twin (right).
Supporting Information Table S7: Results of enrichment testing of putative post‐zygotic mutations in intronic regions
Supporting Information Table S8: Significantly enriched gene clusters in 40‐year‐old twin pair (extended version of Table 2)
Supporting Information Table S9: Significantly enriched gene clusters in the 100‐year‐old twin pair (extended version of Table 2)
Supporting Information Table S10: Comparison of putative post‐zygotic mutations found by Ye et al., (2013)
We looked up the 30 putative mutations that were found by Ye et al. For four loci we found evidence for mosaicism from at least one platform, and three of those were validated with subsequent Sanger sequencing by Ye et al.
Supplementary material: R‐script for simulating erroneously detecting mosaicism due to random sampling

Supp Table S3: Number of putative mosaic mutations, dependent on the minimum difference in allelic ratio at a locus.
Threshold: difference in allelic ratio at a locus. #matching loci = the number of loci where the allelic ratio is highest for the same co‐twin according to both sequencing platforms.
P = the P‐value from testing whether the number of matching loci is more than 50% (standard binomial test). ci_lower and ci_upper are the 95% confidence intervals of the binomial tests.
An allelic ratio difference threshold of 0.25 was chosen for selection of the set of putative post‐zygotic mutations for further analysis.
