# Supplementary material for: A characterization of postzygotic mutations identified in monozygotic twins
Source: Hum Mutat. 2018 Jul 18;39(10):1393–401. doi: 10.1002/humu.23586 (PMC6175188; doi:10.1002/humu.23586)
Supplement: Supplementary file 1 — Supporting Information Table S1: Results of comparing alignment and variant calls from Genalice tools with BWA‐GATK according to best practices. Supporting Information Table S2: Permutation test of putative mosaicism resulting from random sampling heterozygote loci. Heterozygote sites were simulated by sampling from a binomial distribution, with the number of trials defined as the read depth (40X and 13X, respectively). 1000 permutations resulted in a number of loci that would erroneously be identified as a putative post‐zygotic mutation. Supporting Information Table S4: Enrichment testing for within‐pair local clustering of putative post‐zygotic mutations. From the original set of 226945 (40‐year‐old twin pair) and 225010 (100‐year‐old twin pair) heterozygote loci before filtering, we performed 1000 permutations of sampling 1720 (40‐year‐old twin pair) and 1739 (100‐year‐old twin pair) loci. Subsequently we counted the number of times we saw more occurrences of post‐zygotic mutations within a window of distance and compared these to the selected set of putative post‐zygotic mutations. Supporting Information Table S5: Enrichment testing for between‐pair local clustering of putative post‐zygotic mutations. From the original set of 226945 (40‐year‐old twin pair) and 225010 (100‐year‐old twin pair) heterozygote loci before filtering, we performed 1000 permutations of sampling 1720 (40‐year‐old twin pair) and 1739 (100‐year‐old twin pair) loci and counted the number of post‐zygotic mutations found within a specific genetic distance (ranging from 101 basepairs (bp) to 10,000 bp in both twin pairs. Note that the number of post‐zygotic mutations where a post‐zygotic mutation was found nearby in the other twin pair was higher than was expected by chance (indicating genetic hotspots for mosaicism). This enrichment effect is stronger for smaller distances (indicated by the fold change in the last column). Supporting Information Table S6: Number of putative mosaic loci (alleli [file HUMU-39-1393-s001.docx]

# Supplemental Data

# Supplemental Data include 10 tables, 1 script

Supp. Table S1: Results of comparing alignment and variant calls from Genalice tools with BWA-GATK according to best practices.

| Twin | True  positives | False  positives | False  negatives | Precision | Sensitivity | F-measure |
| --- | --- | --- | --- | --- | --- | --- |
| 40-year-old twin A | 1657547 | 22484 | 32730 | 0.9866 | 0.9806 | 0.9836 |
| 40-year-old twin B | 1654005 | 24060 | 31887 | 0.9857 | 0.9811 | 0.9834 |
| 100-year-old twin A | 1655134 | 24884 | 30500 | 0.9852 | 0.9819 | 0.9835 |
| 100-year-old twin B | 1654810 | 24002 | 32406 | 0.9857 | 0.9808 | 0.9832 |

Supp. Table S2: Permutation test of putative mosaicism resulting from random sampling heterozygote loci. Heterozygote sites were simulated by sampling from a binomial distribution, with the number of trials defined as the read depth (40X and 13X, respectively). 1000 permutations resulted in a number of loci that would erroneously be identified as a putative post-zygotic mutation.

|  | Fraction putative mosaic (1000 permutations) | | |
| --- | --- | --- | --- |
| Allelic ratio difference threshold | In 40X data | In 13X data | In both sets simultaneously |
| 0.05 | 0.6423 | 0.8450 | 0.5427 |
| 0.1 | 0.3616 | 0.5572 | 0.2015 |
| 0.15 | 0.1456 | 0.5572 | 0.0811 |
| 0.20 | 0.0637 | 0.3269 | 0.0208 |
| 0.25 | 0.0211 | 0.1686 | 0.0036 |

Supp. Table S4: Enrichment testing for within-pair local clustering of putative post-zygotic mutations. From the original set of 226945 (40-year-old twin pair) and 225010 (100-year-old twin pair) heterozygote loci before filtering, we performed 1000 permutations of sampling 1720 (40-year-old twin pair) and 1739 (100-year-old twin pair) loci. Subsequently we counted the number of times we saw more occurrences of post-zygotic mutations within a window of distance and compared these to the selected set of putative post-zygotic mutations.

|  | 40-year-old twins | | | 100-year-old twins | | |
| --- | --- | --- | --- | --- | --- | --- |
|  | Number of between-twin pairs of post-zygotic mutations in selected set | Mean (sd) of number of within-twin pairs from 1000 random permutations | Local  clustering *p*-value | Number of between-twin pairs of post-zygotic mutations in selected set | Mean (sd) of number of within-twin pairs from 1000 random permutations | Local  clustering  *p*-value |
| 101-500 bp | 11 | 1.37 (1.18) | 0.001 | 11 | 1.313 (1.18) | 0.001 |
| 501-1000 bp | 4 | 1.52 (1.23) | 0.02 | 9 | 1.48 (1.21) | 0.001 |
| 1001-5000 bp | 36 | 9.78 (3.19) | 0.001 | 34 | 9.95 (3.26) | 0.001 |
| 5001 - 10000 bp | 22 | 10.40 (3.32) | 0.001 | 34 | 10.499 (3.24) | 0.001 |

Supp. Table S5: Enrichment testing for between-pair local clustering of putative post-zygotic mutations. From the original set of 226945 (40-year-old twin pair) and 225010 (100-year-old twin pair) heterozygote loci before filtering, we performed 1000 permutations of sampling 1720 (40-year-old twin pair) and 1739 (100-year-old twin pair) loci and counted the number of post-zygotic mutations found within a specific genetic distance (ranging from 101 basepairs (bp) to 10,000 bp in both twin pairs.

Note that the number of post-zygotic mutations where a post-zygotic mutation was found nearby in the other twin pair was higher than was expected by chance (indicating genetic hotspots for mosaicism). This enrichment effect is stronger for smaller distances (indicated by the fold change in the last column).

| Genetic distance | Local clustering *p*-value | Number of between-twin pairs of post-zygotic mutations in selected set | Mean (sd) of number of between-twin pairs from 1000 random permutations | Fold increase of amount of between-twin pairs found in selected set |
| --- | --- | --- | --- | --- |
|  |  |  |  |  |
| 101 - 500 bp | 0.001 | 10 | 1.61 (1.29) | 6.21 |
| 501 - 1000 bp | 0.001 | 7 | 1.79 (1.30) | 3.91 |
| 1001 - 5000 bp | 0.001 | 40 | 12.14 (1.30) | 3.29 |
| 5001 - 10000 bp | 0.001 | 33 | 13.83 (3.74) | 2.37 |
| > 10000 bp | 1 | 25675 | 25675 | 1 |

Supp. Table S6: Number of putative mosaic loci (allelic ratio difference above 0.25) when comparing co-twins of the same sequencing platform (left) and when comparing data from different sequencing platforms of the same co-twin (right).

|  | 13X twin A vs twin B | 40X twin A vs twin B | Twin A 13X vs 40X | Twin B 13X vs 40X |
| --- | --- | --- | --- | --- |
| 40-year-old twins | 1011028 | 1008007 | 732193 | 692373 |
| 100-year-old twins | 1024676 | 994997 | 683775 | 693913 |

Supp. Table S7: Results of enrichment testing of putative post-zygotic mutations in intronic regions

|  | Enrichment tests intronic regions 40-year-old twins | | | |  |  | Enrichment tests intronic regions 100-year-old twins | | | |
| --- | --- | --- | --- | --- | --- | --- | --- | --- | --- | --- |
|  | **A-40X** | **A-13X** | **B-40X** | **B-13X** |  |  | **A-40X** | **A-13X** | **B-40X** | **B-13X** |
| A-40X | x | x | x | x |  | **A-40X** | x | x | x | X |
| A-13X | unenriched | x | x | x |  | **A-13X** | unenriched | x | x | x |
| B-40X | P=1.139E-15 | unenriched | x | x |  | **B-40X** | P=2.380E-14 | unenriched | x | x |
| B-13X | unenriched | unenriched | unenriched | x |  | **B-13X** | unenriched | unenriched | unenriched | x |

Supp. Table S8: Significantly enriched gene clusters in 40-year-old twin pair (extended version of Table 2)

| Category | Term | Count | % | PValue | List Total | Pop Hits | Pop Total | Fold Enrichment | Bonferroni | Benjamini | FDR |
| --- | --- | --- | --- | --- | --- | --- | --- | --- | --- | --- | --- |
| UP_KEYWORDS | Alternative splicing | 658 | 58.08 | 2.43E-22 | 994 | 10587 | 20581 | 1.286865236 | 1.08669066396465E-19 | 1.08669066396465E-19 | 3.44566255135199E-19 |
| UP_SEQ_FEATURE | splice variant | 508 | 44.84 | 7.73E-19 | 973 | 7760 | 20063 | 1.3498484865 | 2.25532393889102E-15 | 2.25532393889102E-15 | 1.38829114590556E-15 |
| UP_KEYWORDS | Polymorphism | 660 | 58.25 | 1.19E-07 | 994 | 12043 | 20581 | 1.1347216405 | 0.000053141 | 2.65708534955555E-05 | 0.000168503 |
| UP_KEYWORDS | Cell junction | 63 | 5.56 | 8.96E-07 | 994 | 675 | 20581 | 1.9324882629 | 0.0004003448 | 0.0001334661 | 0.0012696534 |
| UP_KEYWORDS | Synapse | 40 | 3.53 | 1.74E-06 | 994 | 357 | 20581 | 2.319913881 | 0.0007796183 | 0.0001949616 | 0.0024729355 |
| UP_KEYWORDS | Ion channel | 40 | 3.53 | 2.00E-06 | 994 | 359 | 20581 | 2.3069895697 | 0.0008942689 | 0.0001789178 | 0.0028367629 |
| INTERPRO | IPR000742:Epidermal growth factor-like domain | 30 | 2.65 | 5.35E-06 | 934 | 231 | 18559 | 2.5805778804 | 0.0082109341 | 0.0082109341 | 0.0089183336 |
| GOTERM_BP_DIRECT | GO:0007411~axon guidance | 24 | 2.12 | 5.99E-06 | 858 | 159 | 16792 | 2.9541276334 | 0.0177322693 | 0.0177322693 | 0.010773368 |
| UP_KEYWORDS | EGF-like domain | 29 | 2.56 | 1.26E-05 | 994 | 238 | 20581 | 2.5229063456 | 0.0055951077 | 0.0009346994 | 0.0177891184 |
| UP_SEQ_FEATURE | domain:Ig-like C2-type 5 | 13 | 1.15 | 9.95E-06 | 973 | 55 | 20063 | 4.873755022 | 0.028591396 | 0.0143993689 | 0.0178546775 |
| UP_SEQ_FEATURE | sequence variant | 666 | 58.78 | 1.19E-05 | 973 | 12443 | 20063 | 1.103652016 | 0.0341861782 | 0.0115277716 | 0.0214095273 |
| GOTERM_MF_DIRECT | GO:0005096~GTPase activator activity | 33 | 2.91 | 1.40E-05 | 857 | 279 | 16881 | 2.3298452968 | 0.0133271461 | 0.0133271461 | 0.0219640128 |
| UP_KEYWORDS | Membrane | 425 | 37.51 | 1.73E-05 | 994 | 7494 | 20581 | 1.174235834 | 0.0076983517 | 0.0011034104 | 0.0245012594 |
| UP_KEYWORDS | Metal-binding | 227 | 20.04 | 1.79E-05 | 994 | 3640 | 20581 | 1.2912328366 | 0.0079800857 | 0.0010010108 | 0.025401411 |
| GOTERM_MF_DIRECT | GO:0046872~metal ion binding | 146 | 12.89 | 2.45E-05 | 857 | 2069 | 16881 | 1.3899837181 | 0.0232765702 | 0.0117068098 | 0.0385524066 |
| UP_SEQ_FEATURE | domain:EGF-like 2 | 16 | 1.41 | 2.26E-05 | 973 | 89 | 20063 | 3.7069182535 | 0.0636674763 | 0.0163116517 | 0.0404862887 |

Supp. Table S9: Significantly enriched gene clusters in the 100-year-old twin pair (extended version of Table 2)

| Category | Term | Count | % | PValue | List Total | Pop Hits | Pop Total | Fold Enrichment | Bonferroni | Benjamini | FDR |
| --- | --- | --- | --- | --- | --- | --- | --- | --- | --- | --- | --- |
| UP_KEYWORDS | Alternative splicing | 627 | 55.39 | 7.47E-18 | 970 | 10587 | 20581 | 1.2565777519 | 3.15929468907622E-15 | 3.15929468907622E-15 | 1.05022290081153E-14 |
| UP_SEQ_FEATURE | splice variant | 466 | 41.17 | 1.09E-11 | 946 | 7760 | 20063 | 1.2735879231 | 2.78747508408728E-08 | 2.78747508408728E-08 | 1.92470483995066E-08 |
| UP_KEYWORDS | Polymorphism | 642 | 56.71 | 3.55E-07 | 970 | 12043 | 20581 | 1.1310845758 | 0.0001501786 | 7.50921062211418E-05 | 0.0004992647 |
| KEGG_PATHWAY | hsa04750:Inflammatory mediator regulation of TRP channels | 19 | 1.68 | 1.11E-06 | 343 | 98 | 6910 | 3.9058130541 | 0.0002926824 | 0.0002926824 | 0.0014527791 |
| GOTERM_BP_DIRECT | GO:0035556~intracellular signal transduction | 44 | 3.89 | 1.53E-06 | 827 | 403 | 16792 | 2.2168920521 | 0.0045230488 | 0.0045230488 | 0.0027519605 |
| GOTERM_BP_DIRECT | GO:0051965~positive regulation of synapse assembly | 14 | 1.24 | 7.945E-06 | 827 | 62 | 16792 | 4.5849358349 | 0.0232585596 | 0.0116976979 | 0.0142851513 |
| GOTERM_BP_DIRECT | GO:0043547~positive regulation of GTPase activity | 53 | 4.68 | 1.01E-05 | 827 | 565 | 16792 | 1.9046901585 | 0.0294767252 | 0.0099237297 | 0.0181615744 |
| UP_KEYWORDS | Cell junction | 58 | 5.12 | 1.50E-05 | 970 | 675 | 20581 | 1.8231355479 | 0.0063346983 | 0.0021160406 | 0.0211227577 |
| UP_SEQ_FEATURE | sequence variant | 648 | 57.24 | 1.36E-05 | 946 | 12443 | 20063 | 1.1044718249 | 0.0342650649 | 0.0172818639 | 0.024071408 |
| INTERPRO | IPR011993:Pleckstrin homology-like domain | 42 | 3.71 | 3.16E-05 | 910 | 427 | 18559 | 2.0060169339 | 0.0440029207 | 0.0440029207 | 0.0520987947 |

Supp. Table S10: Comparison of putative post-zygotic mutations found by Ye et al., (2013)

We looked up the 30 putative mutations that were found by Ye et al. For four loci we found evidence for mosaicism from at least one platform, and three of those were validated with subsequent Sanger sequencing by Ye et al.

|  |  |  |  |  |  |  |  |  | |
| --- | --- | --- | --- | --- | --- | --- | --- | --- | --- |
|  | **40-year-old  twin pair** |  |  |  |  |  |  |  | |
| chr | bp | Ref | Twin 1 | Twin 2 | Validated  Sanger | Found with gaMap in  40X data | Found with gaMap in  13X data | Alternative  read ratio  40X data | Alternative  read ratio  13X data |
| 2 | 238496884 | A | A:A | A:G | No | No | No |  |  |
| 2 | 47995854 | A | A:A | A:G | No | No | No |  |  |
| 6 | 150412374 | A | A:A | A:C | No | No | No |  |  |
| 6 | 29905621 | A | A:G | A:A | No | No | No |  |  |
| 6 | 31313556 | G | G:G | A:G | No | No | No |  |  |
| 6 | 57402971 | G | C:G | G:G | No | No | No |  |  |
| 7 | 57284841 | G | G:T | G:G | No | No | No |  |  |
| 7 | 61531167 | C | A:C | C:C | No | No | No |  |  |
| 13 | 19642122 | A | A:G | A:A | No | No | No |  |  |
| 14 | 30525248 | T | A:T | T:T | No | No | Yes |  | 0.36 |
| 16 | 32473534 | A | A:A | A:G | No | No | No |  |  |
| 16 | 33932012 | A | A:A | A:G | No | No | No |  |  |
| 17 | 78660018 | G | A:G | G:G | No | No | No |  |  |
| 18 | 28954823 | T | T:T | G:T | No | No | No |  |  |
| 19 | 56676264 | T | A:T | T:T | No | No | No |  |  |
| 20 | 57786120 | A | A:C | A:A | No | No | No |  |  |
| 20 | 60370175 | A | A:G | A:A | No | No | No |  |  |
|  | **100-year-old  twin pair** |  |  |  |  |  |  |  | |
| Chr | bp | Ref | Twin 1 | Twin 2 | Validated  Sanger | Found with gaMap in  40X data | Found with gaMap in  13X data | Alternative  read ratio  40X data | Alternative  read ratio  13X data |
| X | 49458866 | T | T:T | T:C | Yes | Yes | No | -0.2 |  |
| X | 103014545 | C | C:T | C:C | No* | No | No |  |  |
| 3 | 4772267 | A | A:C | A:A | Yes | Yes | Yes | 0.211 | 0.143 |
| 4 | 3600567 | G | G:T | G:G | No | No | No |  |  |
| 5 | 134136335 | G | G:T | G:G | Yes | No | No |  |  |
| 5 | 165483058 | C | C:T | C:C | No* | No | No |  |  |
| 6 | 164758167 | G | G:G | G:A | Yes | No | No |  |  |
| 11 | 114423117 | T | T:T | T:A | No | No | No |  |  |
| 12 | 8357028 | A | A:A | A:T | No | No | No |  |  |
| 14 | 81921345 | G | G:G | G:A | Yes | No | No |  |  |
| 16 | 83303332 | C | C:T | C:C | Yes | Yes | No | 0.2 |  |
| 20 | 31939491 | C | C:C | C:T | Yes | No | No |  |  |
| 20 | 58727637 | A | A:G | A:A | Yes | No | No |  |  |

Supplementary material: R-script for simulating erroneously detecting mosaicism due to random sampling

# m1=MZ1, m2= MZ2:

Nsimulation <- 843581 # number of tests per permutation, het loci

Npermutation <- 1e3

results <- matrix(nrow = Npermutation, ncol=10)

########

## 40X data

########

i <- 1

j <- 1

start <- Sys.time()

for (i in 1:Npermutation){

m1=rbinom(Nsimulation, 40, 0.5)/40

m2=rbinom(Nsimulation, 40, 0.5)/40

for (diff in seq(0,0.45,0.05)){

results[i,j] <- length(which(abs(m1-m2) > diff )) / Nsimulation # count the number of times there is a difference between co-twins

j <- j + 1

}

}

lablist <- as.vector(seq(0,0.45,0.05))

colnames(results) <- as.character(seq(0,0.45,0.05))

########

## 13X data

########

results13 <- matrix(nrow = Npermutation, ncol=10)

i <- 1

j <- 1

start <- Sys.time()

for (i in 1:Npermutation){

m1=rbinom(Nsimulation, 13, 0.5)/13

m2=rbinom(Nsimulation, 13, 0.5)/13

for (diff in seq(0,0.45,0.05)){

results13[i,j] <- length(which(abs(m1-m2) > diff )) / Nsimulation # count the number of times there is a difference between co-twins

j <- j + 1

}

}

lablist <- as.vector(seq(0,0.45,0.05))

colnames(results13) <- as.character(seq(0,0.45,0.05))

# Plot

plot(logresults[1,], type = "l", xlab = "Difference in allelic ratio", ylab="Percentage of false positives (log)", xaxt='n', main="Percentage of false positive mosaics \ndue to random sampling", lwd=2)

text(seq(1, 10, by=1), par("usr")[3] - 0.05, labels = lablist, srt = 45, pos = 1, xpd = TRUE)

for (i in 2:nrow(logresults)){lines(logresults[i,], col="yellow", lwd=2}

grid(nx = NULL, ny = NULL, col = "lightgray", lty = "dotted", lwd = par("lwd"), equilogs = TRUE)

abline(a = log10(0.05), b = 0, col=rgb(1,0,0,0.3), lwd=2, lty=2)

#grid(col = "lightgray", lty = "dotted", lwd = par("lwd"), equilogs = TRUE)

abline(v = 1, lty = "dotted",col="lightgray"); abline(v = 3, lty = "dotted",col="lightgray")

abline(v = 5, lty = "dotted",col="lightgray"); abline(v = 7, lty = "dotted",col="lightgray")

abline(v = 9, lty = "dotted",col="lightgray"); abline(v = 11, lty = "dotted",col="lightgray")

lines(logresults13[1,], type = "l", lwd=2)

text(seq(1, 10, by=1), par("usr")[3] - 0.05, labels = lablist, srt = 45, pos = 1, xpd = TRUE)

for (i in 2:nrow(logresults13)){lines(logresults13[i,], col="red", lwd=2)}

grid(nx = NULL, ny = NULL, col = "lightgray", lty = "dotted", lwd = par("lwd"), equilogs = TRUE)

abline(a = log10(0.05), b = 0, col=rgb(1,0,0,0.3), lwd=2, lty=2)

#grid(col = "lightgray", lty = "dotted", lwd = par("lwd"), equilogs = TRUE)

abline(v = 1, lty = "dotted",col="lightgray"); abline(v = 3, lty = "dotted",col="lightgray")

abline(v = 5, lty = "dotted",col="lightgray"); abline(v = 7, lty = "dotted",col="lightgray")

abline(v = 9, lty = "dotted",col="lightgray"); abline(v = 11, lty = "dotted",col="lightgray")

legend("bottomleft",inset=.05, title="Read depth", c("13X","40X"), fill=c(rgb(1,0,0),rgb(1,1,0)), horiz=TRUE)
